# Craving Ravens: Individual ‘haa’ Call Rates at Feeding Sites as Cues to Personality and Levels of Fission-Fusion Dynamics?

**DOI:** 10.12966/abc.08.04.2014

**Published:** 2014-08-01

**Authors:** Georgine Szipl, Thomas Bugnyar

**Affiliations:** 1 University of Vienna

**Keywords:** Food-associated calls, Fission-fusion, Personality, Common ravens, *Corvus corax*

## Abstract

Common ravens aggregate in large non-breeder flocks for roosting and foraging until they achieve the status of territorial breeders. When discovering food, they produce far-reaching yells or ‘haa’ calls, which attract conspecifics. Due to the high levels of fission-fusion dynamics in non-breeders’ flocks, assemblies of feeding ravens were long thought to represent anonymous aggregations. Yet, non-breeders vary in their degree of vagrancy, and ‘haa’ calls convey individually distinct acoustic features, which are perceived by conspecifics. These findings give rise to the assumption that raven societies are based on differential social relationships on an individual level. We investigated the occurrence of ‘haa’ calling and individual call rates in a group of individually marked free-ranging ravens. Calling mainly occurred in subadult and adult females, which showed low levels of vagrancy. Call rates differed significantly between individuals and with residency status, and were correlated with calling frequency and landing frequency. Local ravens called more often and at higher rates, and were less likely to land at the feeding site than vagrant birds. The results are discussed with respect to individual degrees of vagrancy, which may have an impact on social knowledge and communication in this species.

In a variety of species, the distribution of food has strong impacts on group size (e.g., Dechmann, Kranstauber, Gibbs, & Wikelski, 2010; Fortin & Fortin, 2009; Foster et al., 2012; Majolo et al., 2009), and group structure (e.g., Barton, Byrne, & Whiten, 1996; Dyer, Croft, Morrell, & Krause, 2009; Morand-Ferron & Quinn, 2011; Watts, 1985). This so-called group effect (Elgar, 1989) grants increased foraging efficiency with increasing group size due to shared vigilance, but also an increase in competition over food. The degree of competition depends, amongst others, on the quality and quantity of the food that has to be shared (Elgar, 1989). The extent to which animals’ social groups vary in their size and composition in space and time - the degree of fission-fusion dynamics - is supposed to influence not only foraging, but also several aspects of cognitive, communicative, and social abilities (Aureli et al., 2008).

In the feeding context, individuals that are part of highly dynamic societies have to pay attention to social cues that could inform about changes in dominance hierarchies while absent, or shifts in feeding patches. One way to gather information on the latter one may be through acoustic means. Many animals signal the discovery of food by uttering acoustic signals. These food-associated calls may provide diverse pieces of information. First and foremost, they can signal the presence of food (e.g., Evans & Evans, 1999). If acoustic signals refer to an object in the environment, and in receivers elicit a response as if they would see the actual object themselves, these signals are termed functionally referential (Evans, 1997; Macedonia & Evans, 1993; Marler, Evans, & Hauser, 1992). From hearing food-associated calls, receivers would also gain information on the approximate location of the food source (e.g., Dittus, 1984). Some primate species use several acoustically distinct food-associated calls when encountering different types of food (Hauser & Marler, 1993; Slocombe & Zuberbühler, 2005). However, most species employ only one type of food-associated call, and information on the type of food (Elowson, Tannenbaum, & Snowdon, 1991), or its quality and amount (Caine, Addington, & Windfelder, 1995), is provided through differential rates in calling. In general, call rate increases with better or more or preferred food types, suggesting that arousal provokes these changes (reviewed in Briefer, 2012). Only few studies could show intentional signaling so far (Chapman & Lefebvre, 1990; Schel, Machanda, Townsend, Zuberbühler, & Slocombe, 2013), indicating that in most species, food-associated calls are not under voluntary control (reviewed in Clay, Smith, & Blumstein, 2012). Individual characteristics in food-associated calls have received little attention. This is surprising, because identifying the individual that signals food may benefit the receiver in various ways. Receivers could integrate social information gathered via direct or indirect interactions, as well as kinship, sex, or rank of the calling individual (Clay, et al., 2012), and thereby avoid costly agonistic encounters over food, or gain easier access to it.

Food-associated calls in Common ravens (*Corvus corax*), termed yells or ‘haa’ calls, are loud and far-reaching calls uttered at feeding sites (Heinrich, 1988b). These calls are primarily given by young non-breeding ravens when encountering an ephemeral food source such as a carcass or kill of a large mammal (Heinrich, 1988b). ‘Haa’ calling attracts conspecifics and significantly contributes to raven assemblies at large food sources (Heinrich & Marzluff, 1991). Functionally, ‘haa’ calling may pay off because forming foraging groups increases the chances for an individual raven to overcome the food defense of dominant territorial breeders or dangerous predators (Heinrich, 1988b). Moreover, attracting others to feeding sites offers the opportunity to engage in alternative strategies such as kleptoparasitizing successful conspecifics or pilfering their food caches (Bugnyar & Kotrschal, 2002). On the proximate level, ‘haa’ call rates vary with the in-/ability to access food as well as with its quality and quantity. In experiments, wild ravens called most when food was placed in a bucket next to a known feeding site but quickly stopped calling when it was put onto the feeding site and thus became available to the birds (Bugnyar, Kijne, & Kotrschal, 2001). Furthermore, ravens called most when seeing high quality food, possibly reflecting their motivational state to feed. ‘Haa’ call rates thus seem affected by hunger level and the inability to access food. Yet, callers appear to be sensitive to the audience, as call activity was reduced with an increasing number of approaching ravens (Bugnyar et al., 2001). Taken together, those findings met the production criterion for functionally referential signals, denoting the presence of food. Whether receivers utilize this information has yet to be shown.

To our knowledge, all studies on food-associated calls in wild ravens have been conducted at the group level, probably due to constraints in marking and monitoring individual birds. When temporarily brought into captivity, however, ravens were reported to display individual differences in ‘haa’ calling rate (Marzluff & Heinrich, 1991). Moreover, recent experiments showed that the acoustic structure of ‘haa’ calls contains individually distinct features, which can be perceived by conspecifics (Boeckle, Szipl, & Bugnyar, 2012). These findings raise the possibility that ravens utilize information about individual callers. Indeed, raven non-breeder aggregations may favor this ability as they appear to be structured on different levels. On one hand, we find gradual degrees of fission-fusion dynamics, with some birds developing strong preferences for particular locations, whereas others remain highly vagrant (Braun & Bugnyar, 2012; Dall & Wright, 2009). Thus, individuals can be categorized by their residency status. On the other hand, many birds interact repeatedly over extended time periods, offering the opportunity for individual recognition and the formation of differential individual social relationships (Braun, Walsdorff, Fraser, & Bugnyar, 2012).

We here investigated ‘haa’ call rates in a group of free-ranging ravens on an individual level while considering fission-fusion dynamics. We observed individually marked birds foraging at the enclosures of zoo animals (brown bears and wild boars) in the Austrian Alps. We were interested in the calling behavior right before the feedings (when ravens could see but not access the food) and during the feedings (when ravens descend inside the enclosures to snatch food from the zoo animals). Specifically, we focused on the features of individual callers like their sex and age class and, notably, their experience with the local situation, reflected by the amount of time spent at this location (residency status). We speculated that this fine-scaled approach may offer a new perspective on ‘haa’ calling at feeding sites. We expected that calling may not solely be a function of hunger and age, but also vary with factors like sex, individual degree of fission-fusion, and thus individual knowledge of specific environmental circumstances. Alternatively, call rates might reflect a stable individual characteristic and would be expressed by certain individuals across contexts.

## Method

### Study site and free-ranging population of Common ravens

The study took place at the area of the Cumberland Gamepark (47°48′N, 13°57′E), a local Gamepark near Gruenau im Almtal, upper Austria. This area holds a group of free-ranging Common ravens that use the Gamepark for foraging year-round. The ravens are habituated to the presence of humans and have been under constant observation since 2007. Since then, in the course of on-going long-term studies, ravens are captured and individually marked (see Braun & Bugnyar, 2012 for details). During the marking procedure, blood samples are taken to determine sex. Age was determined by the color of the inner beak (Heinrich & Marzluff, 1992). The color usually turns from pinkish red to black within the first three years. Because some of the marked individuals had completely black oral cavities at trapping and marking, those birds were categorized as adults (older than 3 years). Birds with partially black oral cavities were categorized as subadults (likely 1 to 3 years of age), and birds with primarily pink oral cavities were classified as juveniles in their first year.

### Feeding procedure

Data were collected on 37 days between June and November 2010. Two consecutive feedings were conducted in the morning (0700-0900 o’clock) at the enclosures of bears (*Ursus arctos*) and wild boars (*Sus scrofa*). The diet of both bears and wild boars is comparable and comprises a mixture of fruits, vegetables, and bread. Additionally, bears are fed moderate amounts of fish or chicken, and wild boars are occasionally provided with kitchen leftovers and entrails. Before each feeding, the food was put next to the enclosure so that it was visible to the ravens, but not accessible for a total of 10 minutes. During this 10-minute pre-feeding phase, marked birds perched in the trees were identified, and focal observations on calling individuals were conducted (see below). After this pre-feeding phase, the food was provided to the zoo animals, and the identity of all ravens landing (descending on the ground inside the enclosure) within the first two minutes after the food was accessible was noted. After the food was consumed, which lasted 20 to 30 minutes, the second feeding was conducted in the same way. Feeding location (the bear or the wild boar enclosure) and feeding order (the first or the second feeding) were randomized.

### Occurrence of calling

For each marked bird visible in the trees inside and surrounding the enclosures, we noted their identity and whether they uttered ‘haa’ calls or not during the 10 minutes prior to feeding. In total, 1122 sightings of 56 marked individuals were obtained. Out of 1122 cases, 135 (12%) were calling events. Only 11 birds (19.6%) produced ‘haa’ calls during the study period. Out of the 11 calling birds observed, eight birds were females (72.7%), and only three birds were males (Table 1).

### Focal observations

If a marked individual was detected producing ‘haa’ calls during the 10-minute phase prior to feeding, focal observations were conducted using binoculars and a digital voice recorder (Olympus VN-3100). During the focal observation, each ‘haa’ call produced by the focal individual was annotated and later counted up from the voice recorder. The focal observations stopped after five minutes or earlier if the focal bird flew off or moved out of sight. Minimum sampling time was one minute. One to three individuals were sampled before each feeding (mean ± SD = 1.29 ± 0.14).

### Residency status

Marked individuals were sighted between 2 and 54 times during the course of the study. Based on the percentage of sightings at feedings within the observation period, residency status was calculated for each marked individual during that period. Ravens that were present on over 60% (> 22 days) of the time were categorized as residents, birds present between 30 to 60% of the time (11-21 days) were termed visitors, and birds observed on less than 30% of the time (< 11 days) were considered vagrants. The number of birds present per day per enclosure ranged from 10 to 34 marked individuals (mean ± SD = 30.32 ± 14.92).

### Statistics

For the occurrences of ‘haa’ calling (1122 cases), Generalized Linear Mixed Models (GLMMs) were fitted using a binomial family with a logit link function. Individual identity of the birds was used as a random effect to account for unbalanced repeated sampling. Fixed effects included feeding order (first or second), location (bear or wild boars enclosure), sex, age class, and residency. Due to singularities in the levels of fixed effects, the full model did not contain all possible interactions (see Table 2).

To investigate individual ‘haa’ call rates, individuals observed less than four times were excluded from analysis due to low sample sizes. Unfortunately, all males met this criterion. For the remaining six females, individual ‘haa’ call rates were calculated as the total number of calls divided by the total time observed and multiplied by 60 to obtain calls per minute for each focal observation (63 cases). Furthermore, calling frequency (times observed calling/total number of observations*100) and landing frequency (times observed landing/total number of observations*100) of each focal bird were calculated. In addition to nonparametric tests, GLMMs for individual call rates were fitted using a Poisson distribution with a log link function.

To investigate the relationship between individual call rates, calling frequency, and landing frequency, the focal individuals’ identity was entered as random factor to account for repeated measures, and landing and calling frequency as well as their two-way interaction were used as fixed factors. To analyze the influence of individuality and residency onto call rates, two models were fitted and a likelihood ratio test was performed to compare the two models. As random factor, sampling day and a crossed term that included location and feeding order was entered. Fixed factors were focal individual or residency.

Aside from the models investigating call rates with respect to individuality and residency, the final model selections were based on Akaike’s Information Criterion (AICc) values. Models were ranked using several measures of strength of evidence (Burnham, Anderson, & Huyvaert, 2011). As the difference in AICc (ΔAICc) is better suited to determine the best model, the difference was calculated by subtracting the lowest AICc from each AICc. Further, the relative likelihood (exp (−0.5/ΔAICc)) and the probability, or Akaike weight (relative likelihood/sum of all relative likelihoods) were computed (Table 2). These measures were used to directly compare two models and to judge their strength of the evidence (Burnham, et al., 2011). Only models with ΔAICc less than 2 are presented (Table 3).

GLMMs were fitted by maximum likelihood (glmerMod) using the lme4 package (Bates, Maechler, Bolker, & Walker, 2013) in R (R Core Team, 2013). Nonparametric tests were performed using SPSS 19. Significance levels were set to 0.05, and 2-tailed results are shown.

## Results

### Occurrence of calling

In the course of the study, we had a total of 1122 sightings of 56 marked individuals before the feedings of bears and wild boars. In 135 (12%) of these sightings, ravens were observed to engage in ‘haa’ calling, whereby only 11 out of the 56 birds (19.6%) contributed to these calling events (Table 1). If we assume the likelihood of calling and non-calling to be 50:50, ‘haa’ calling was observed significantly less often than expected by chance (Chi-Square: χ^2^ = 646.973, *df* = 1, *p* < 0.001).

**Figure 1:**
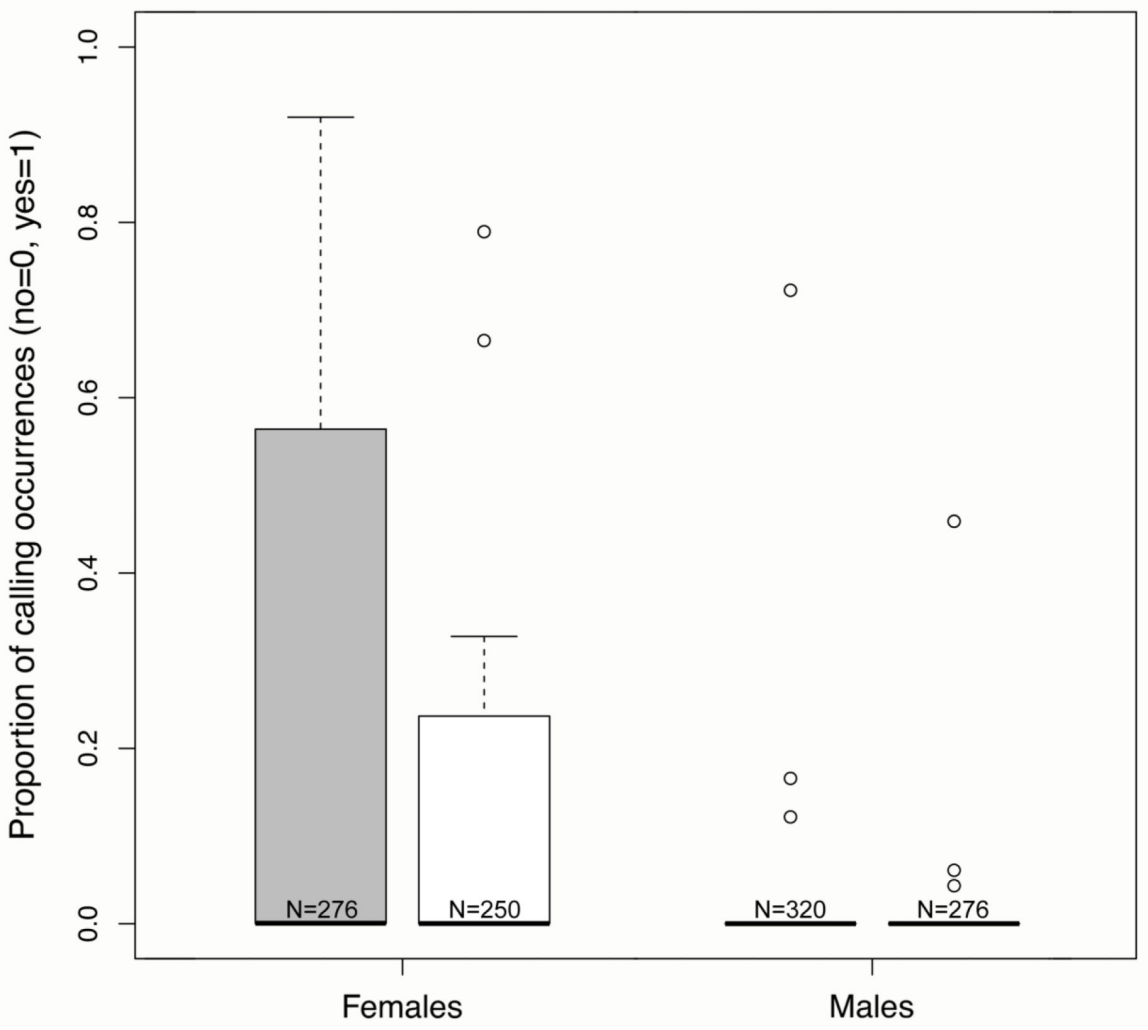
Proportion of calling occurrences (0 = no calling; 1 = calling) before the first (grey bars) and the second feeding (white bars) for female and male ravens. Values are estimated means derived from the GLMM, and are controlled for fixed and random effects. Whiskers represent the minimum and maximum, bold lines the median of all data, and circles indicate outliers. N denotes the number of cases for each group.

There was a marginal decrease in the number of calling events when the wild boars were fed first and the bears second (McNemar test: *N* = 37, *p* = 0.063), but not the other way around. The final model on the occurrence of calling as a binomial response revealed a significant influence of feeding order (GLMM: pair-wise comparison: ß = −1.121, SE = 0.310, *z* = −3.616, *p* = 0.0003), with more calling events before the first feeding. Based on the strength of evidence provided by the model likelihoods and probabilities (Table 2a), the models including location, or sex (Figure 1) in combination with feeding order are also highly qualified to explain the data.

### Who is calling?

In the cases in which calling occurred (*N* = 135), there was a strong bias towards females (8 females, 3 males; Chi-Square: χ^2^ = 53.519, *df* = 1, *p* < 0.001), and significantly more observations on subadult and adult individuals than juveniles (3 subadults, 7 adults, 1 juveniles; χ^2^ = 26.800, *df* = 2, *p* < 0.001). Most importantly, individuals that were observed calling did not call on every occasion. When correcting for the number of sightings, individuals called in 9.5% to 87.0% of feeding events.

Out of the 11 calling birds, residents and visitors produced ‘haa’ calls most frequently, and differed significantly in the frequency of observed calling events from vagrant ravens that only rarely visited the study area (Chi-Square: Residents vs. Vagrants: χ^2^ = 32.014, *df* = 1, *p* < 0.001; Visitors vs. Vagrants: χ^2^ = 39.286, *df* = 1, *p* < 0.001). All focal birds, their residency status, sex, age class, and times observed calling are summarized in Table 1.

### Individual call rates

**Figure 2:**
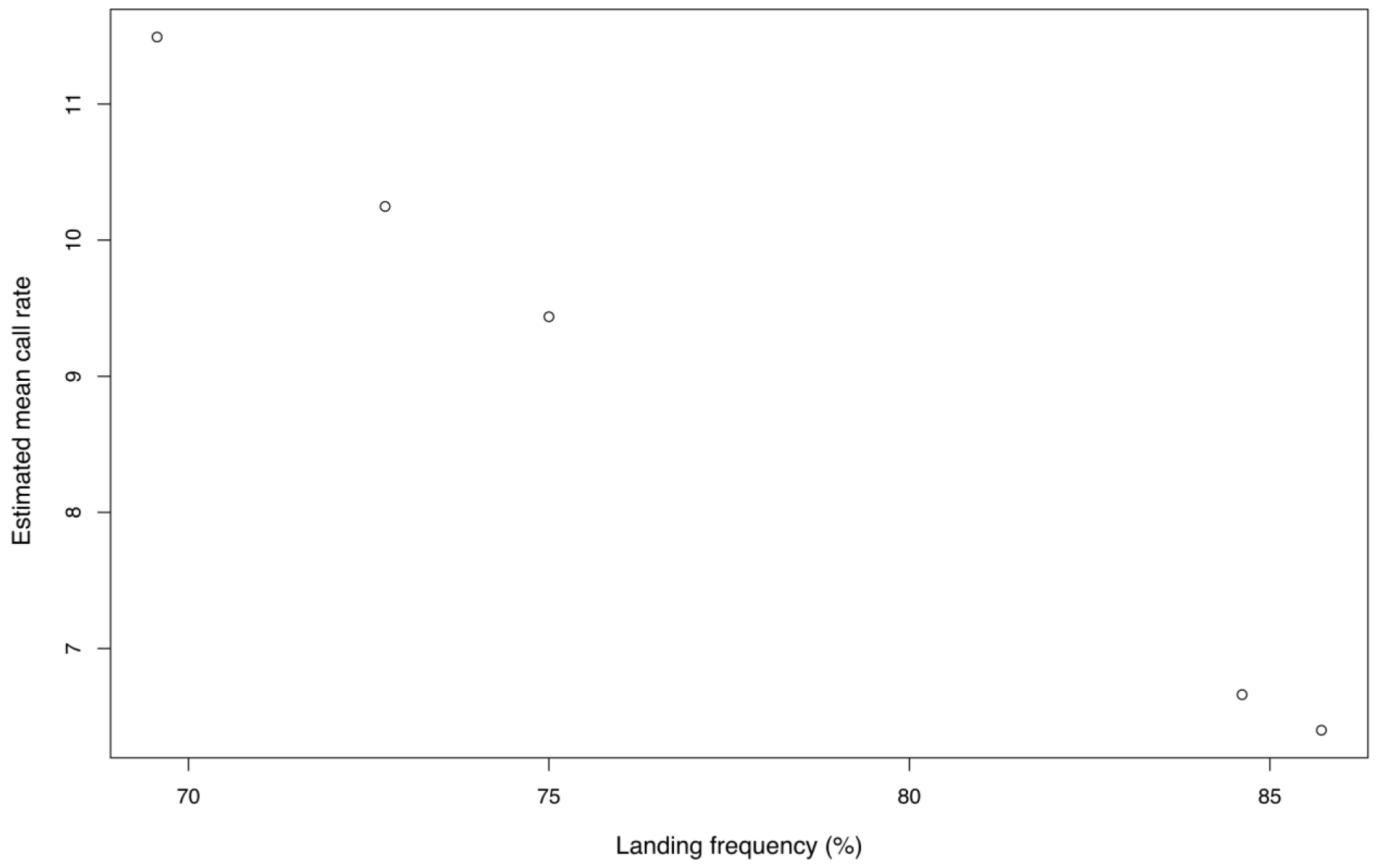
Estimated mean call rate and landing frequency of six females, showing the negative correlation between individual call rates and landing frequencies. Values are taken from the GLMM and corrected for repeated sampling.

Analyses on six females revealed a negative correlation between individual call rate and landing frequency (Spearman’ rho: r_s_ = −0.899, *p* = 0.015), i.e., birds that were more likely to land in an enclosure had lower call rates. A model investigating the influence of individual calling and landing frequency onto call rates confirmed this result (Table 2b). The final model included landing frequency (GLMM: pair-wise comparison: ß = −0.036, SE = 0.006, *z* = −5.833, *p* < 0.0001) and showed a negative relationship between call rate and landing frequency (Figure 2).

**Figure 3:**
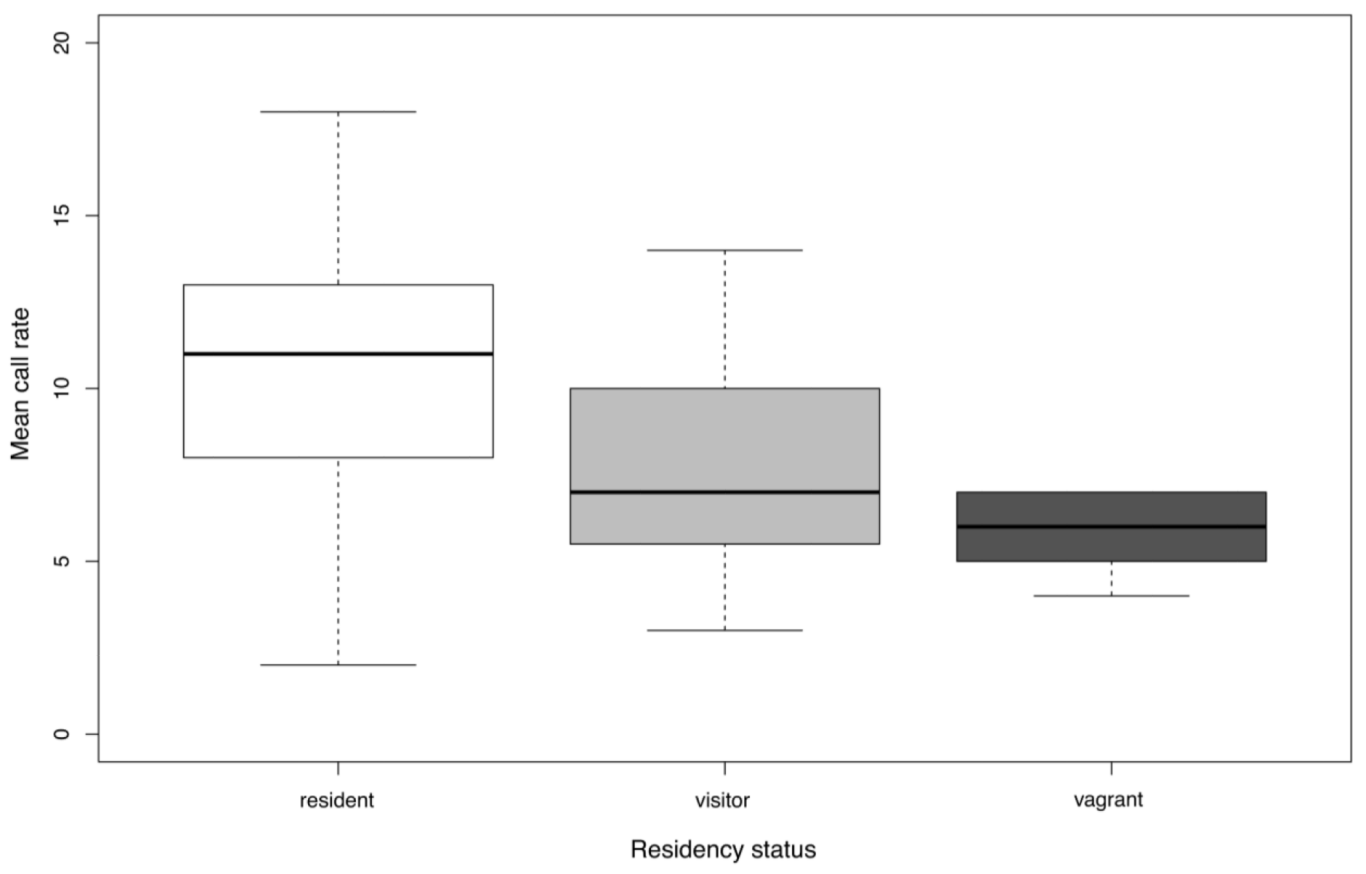
Differences in mean call rate with respect to residency status of six females. Boxplots show interquartile ranges with median (bold line), whiskers represent the minimum and maximum of all the data.

Call rates of six females varied strongly with residency status (Figure 3). Residents (*N* = 2) had a mean call rate of 10.85 ± 3.6 (SD) calls per minute, while visitors (*N* = 3) called at a mean rate of 8.22 ± 4.56. The vagrant female (*N* = 1) had the lowest call rate with a mean at 5.78 ± 1.28 call per minute. Conversely, the two resident females had the lowest mean landing frequency (70.43% ± 1.99%), whereas the three visiting females landed 80.65% ± 5.89% of the time, and the vagrant female even 85.71% of all observations.

The two models investigating the influence of individuality and residency onto call rates showed that both residency and individuality were highly significant factors (Table 2c). However, the likelihood ratio test revealed that the model including individual identity explained variations in call rates significantly better than residency status (Chi-Square: χ^2^ = 16.455, *p* = 0.0009).

Finally, no differences were found in individual call rates at different enclosures (Mann-Whitney U test: bears vs. wild boars: U_1st feeding_ = 192.0, *p* = 0.776; U_2nd feeding_ = 16.0, *p* = 0.131), with feeding order (Wilcoxon signed-rank test: 1st vs. 2nd feeding: Z_bears_ = −1.461, *p* = 0.144; Z_wild boars_ = −0.730, *p* = 0.465), or in the combination of location and feeding order (Z_bears1st/wild boars2nd_ = −0.944, *p* = 0.345; Z_wild boars1st/bears2nd_ = −1.461, *p* = 0.144).

## Discussion

The investigation of the occurrence of ‘haa’ calling events before the feedings of zoo animals showed that only a minority of the ravens present around the enclosures contributed to the calling, and they did so at different rates. Feeding order had a significant influence on calling events, with ravens calling more often before the first feeding, especially when the bears were fed first. Calling birds were mainly females, and experienced with the local situation, as residents and visitors called more often than vagrants. The rates at which birds called, however, did not differ with feeding order and location, but appeared to be an individual characteristic that is influenced by experience (residency status). Further, call rate and landing frequency were correlated: birds that called at higher rates were less likely to land at the feeding site after the food was made available to them.

### Occurrence of calling

Our findings are in line with those of previous studies showing that Common ravens produce ‘haa’ calls in expectation of food (Bugnyar et al., 2001; Heinrich, 1988b; Heinrich & Marzluff, 1991). Moreover, the fact that ravens called ‘haa’ most often at the first feeding of a day supports the assumption of hunger level being the driving motivation behind ‘haa’ calling occurrences. However, we found that only a fraction of all birds present at the feeding ever produced ‘haa’ calls. The majority of birds were never observed calling, whereas some individuals were observed frequently. This can hardly be interpreted as most ravens not being hungry, because they do competitively participate at the feeding and try to carry off food for caching. One possible explanation is that a certain threshold in hunger level must be exceeded before ravens start ‘haa’ calling at feeding sites. This may be reflected also in our finding that those individuals observed calling did not do so on every occasion but in 9.5% to 87.0% of the time. Differences in rank may reduce subordinates’ chances to obtain enough food at the feedings (e.g., Forrester, 1991; Vogel, 2005). Therefore, some ravens would stay hungry more often, and call more frequently. However, 9.5% to 87.0% is a rather wide range for the occurrences of ‘haa’ call if the only reason to call would be hunger level. Another possibility is that, additionally to hunger level, some individuals may be better able to suppress the urge to call than others. Ravens are renowned for their ability to control intentions at foraging (Bugnyar & Heinrich, 2005; Bugnyar & Kotrschal, 2002) and in delay of gratification experiments they are able to wait for food up to five minutes (Dufour, Wascher, Braun, Miller, & Bugnyar, 2012). However, impulse or self-control, tested with food as reward, revealed negative correlations with metabolic rate in pigeons, rats, and humans (Tobin & Logue, 1994). On these grounds, the hungrier a raven would be, the more likely it might be to signal its hunger by calling ‘haa’, irrespective of its general ability to suppress current motivational states for future rewards.

### Individual call rates

As neither the location nor feeding order had an influence on individual call rates, apparently, call rates differ irrespective of hunger level. Individual call rates were negatively correlated with landing frequency, indicating that individuals that called at high rates were more hesitant to land at the feeding site. On the contrary, most individuals that called at lower rates quickly descended. The same was true when individuals were grouped for their residency status: vagrants, that joined the daily feedings less than 30% of the time, had the lowest call rates, and landed most frequently within the focal observations. The models investigating the influence of individuality and residency onto call rates revealed individuality to be more powerful to explain variations in call rates. However, these patterns possibly reflect individual differences in behavior, i.e., behavioral syndromes defined as temporarily and contextually stable differences between individuals (Sih, Bell, & Johnson, 2004). Individual personality may influence dominance rank, which in turn may affect feeding success (David, Auclair, & Cézilly, 2011). In line with this, shy ravens would be lower in rank, and thus stay hungry more often, which could influence their call rates at feeding sites. Also, proactivity is closely linked to exploratory and foraging behavior, and boldness in novel environments (reviewed in Sih et al., 2004). Proactive individuals thus are more prone to take risks, explore their environment faster, and also disperse further, as was shown in fish (Cote, Fogarty, Weinersmith, Brodin, & Sih, 2010; Fraser, Gilliam, Daley, Le, & Skalski, 2001) and birds (Dingemanse, Both, van Noordwijk, Rutten, & Drent, 2003). Thus, residency status in ravens may reflect individual differences in personality, with proactive individuals being more vagrant and less neophobic. Ravens are renowned to be highly neophobic birds, and especially in the feeding context, are hesitant to approach food in unfamiliar situations (Heinrich, 1988a; Kijne & Kotrschal, 2002). Neophobia experiments conducted on the ravens of the very study population confirmed that the presentation of novel objects close to the feeding sites lead to an increase in ‘haa’ call rates before feedings (unpubl. data). This is why we would have expected vagrants that are hardly accustomed to our study site to show more neophobia, be more hesitant to land and more likely to call ‘haa’ than the resident and visiting ravens that have familiarized themselves with the local situation and feeding sites already. However, as we found the exact opposite, we may consider individual personality to act on levels of fission-fusion dynamics in ravens. Our observation period was rather short to judge whether call rates are indeed stable over time, but we have some indication that individual call rates varied with different situations (feeding at a potentially dangerous place, the bear enclosure vs. feeding at the wild boars) in the same context (feeding).

Alternatively, differences in calling behavior with respect to individuality and/or residency status may also reflect differing levels of social knowledge within non-breeder groups. Resident birds, which were present over 60% of the observational period, had the highest call rates and called most often throughout the study. These residents should also have a better knowledge of the individuals within the local population than visitors or vagrants. Ravens are highly attracted by ‘haa’ calls of conspecifics (Heinrich, 1988b), and we recently got indications that receivers respond differentially to ‘haa’ calls of familiar and unfamiliar individuals (Szipl et al., submitted). Ravens not only approached playbacks of familiar females most often, they also appeared to take relative rank and affiliation status to the caller into account, indicating detailed knowledge of conspecifics and their social relationships. In accordance with this, resident birds may call more and at higher rates in expectation of particular conspecifics to arrive. This would coincide with residents not landing immediately, but presumably waiting for other ravens to arrive at the feeding site before they descend. Aside from vigilance or dilution, group size and composition might determine when resident ravens are ready to land at the feeding site. Group size was reported to have an influence on feeding success in non-breeder ravens (Marzluff & Heinrich, 1991), mainly because higher numbers of birds are needed to overcome territorial defense. However, at commonly used feeding sites like our zoo enclosures that are outside of breeding territories, coordination with specific individuals might be more crucial than being part of a group per se. Ravens show dependent rank (Braun & Bugnyar, 2012; Gwinner, 1964) and may form same-sex alliances even with non-kin (Fraser & Bugnyar, 2010, 2012). Thus, waiting for social allies to arrive may help to overpower some dominants that monopolize food. Yet, it needs to be tested whether ravens use their social allies to gain better access to food.

## Conclusion

We conclude that, as previously reported, hunger is one factor that influences ‘haa’ calling in ravens. However, additional factors may determine whether, and at which rates, individuals call ‘haa’ at the sight of food. These factors may include personality traits such as boldness/shyness that could account for dominance rank, and thereby for access to food, and impulse control. Further, individual degrees of vagrancy, and thus social knowledge, could be affected by proactivity or high exploratory behavior. Although we cannot provide a single explanation for differing call rates and frequencies at this point, our findings may set the scene for future studies designed to disentangle the causes and effects of personality and levels of fission-fusion dynamics onto communication at feeding sites in wild ravens.

## Figures and Tables

**Table 1:** Individuals with Residency Status, Sex, Age Class, and Times Observed Calling ‘haa’ Throughout the Observation Period

Individual	Residency Status	Sex	Age Class	Times Observed Calling
**Bi**	**resident**	**female**	**subadult**	**47**
**Go**	**resident**	**female**	**adult**	**5**
Ky	resident	female	adult	0
MF	resident	female	adult	0
Pu	resident	female	adult	0
Qu	resident	female	adult	0
Ut	resident	female	adult	0
ZaF	resident	female	adult	1
Fo	resident	male	subadult	0
Ki	resident	male	subadult	5
M	resident	male	adult	0
Si	resident	male	adult	0
Yo	resident	male	subadult	0
Za	resident	male	adult	0
Al	visitor	female	subadult	0
**Hm**	**visitor**	**female**	**adult**	**12**
Id	visitor	female	subadult	0
Ka	visitor	female	adult	0
**La**	**visitor**	**female**	**subadult**	**14**
MrF	visitor	female	adult	0
Su	visitor	female	adult	0
**Ti**	**visitor**	**female**	**adult**	**20**
Tr	visitor	female	subadult	0
Ca	visitor	male	subadult	0
Cp	visitor	male	adult	0
Gl	visitor	male	subadult	0
HF	visitor	male	adult	0
Ho	visitor	male	adult	0
Kl	visitor	male	subadult	0
Kr	visitor	male	adult	0
Lf	visitor	male	subadult	0
Mq	visitor	male	juvenile	18
Mr	visitor	male	adult	2
Mt	visitor	male	subadult	0
Pa	visitor	male	subadult	0
Pj	visitor	male	subadult	0
Se	visitor	male	adult	0
Sh	visitor	male	subadult	0
Bs	vagrant	female	subadult	0
Di	vagrant	female	adult	0
Fk	vagrant	female	adult	0
**Ge**	**vagrant**	**female**	**adult**	**9**
Mb	vagrant	female	subadult	0
Mi	vagrant	female	adult	0
Mo	vagrant	female	adult	0
Ou	vagrant	female	subadult	0
Sl	vagrant	female	adult	2
Zo	vagrant	female	subadult	0
Ad	vagrant	male	subadult	0
Dd	vagrant	male	subadult	0
El	vagrant	male	subadult	0
Fn	vagrant	male	juvenile	0
Mn	vagrant	male	adult	0
Od	vagrant	male	subadult	0
Ru	vagrant	male	subadult	0
Zu	vagrant	male	subadult	0

*Note*. Individuals used in call rate analysis are indicated in bold type.

**Table 2:** Model Ranking Based on Relative Likelihoods and Akaike Weights Calculated from ΔAICc Values for Response Variables

Response Variable	Random Effects	Distribution	Model	AICc	ΔAICc	Relative Likelihood	Akaike Weight
a) Calling yes/no	focal individual	Binomial	Location * Feeding order * Sex + Age class + Residency (Full model)	383.4205	11.620	0.003	0.001
			Feeding order (Final model)	371.7031	0	1.000	0.193
			Feeding order + Sex	372.4543	0.751	0.687	0.132
			Location + Feeding order	372.5212	0.818	0.664	0.128
			Location + Feeding order + Sex	373.2710	1.568	0.457	0.088
			Location * Feeding order	373.5899	1.887	0.389	0.075
b) Call rate	focal individual	Poisson	Calling frequency * Landing frequency	339.5723	2.480	0.289	0.136
			Calling frequency + Landing frequency	337.6379	0.546	0.761	0.357
			Calling frequency : Landing frequency	346.1645	9.073	0.011	0.005
			Calling frequency	342.3514	5.260	0.072	0.034
			Landing frequency	337.0919	0	1.000	0.469
c) Call rate	sampling day + location : feeding order	Poisson	Residency	353.0033			
			Focal	342.5482			

*Note*. (+) indicate single factors added in the models; (:) indicate interactions between factors; (*) indicate the cross between factors (factors and their two-way interaction)

a) Calling occurrence (yes/no)

b) Call rate as a function of calling frequency and landing frequency

c) Call rate with respect to residency and individual identity, showing AICc values for the two competing models

**Table 3:** Coefficients of Full and Final Models, and Comparably Best Fitting Models, with Pairwise Comparisons for Levels of Each Fixed Effect in the Model

Response Variable	Model	Fixed Effects	Estimate	SE	*z* Value	*p*	Sig. Codes
a) Calling (yes/no)	Location * Feeding order * Sex + Age class + Residency (Full model)	(Intercept)	−3.708	1.528	−2.427	0.0152	*
		Location (Bears vs. Wild boars)	−0.835	0.472	−1.769	0.0768	+
		Feeding order (First vs. Second)	−1.101	0.668	−1.647	0.0995	+
		Sex (Female vs. Male)	−3.870	2.258	−1.714	0.0865	+
		Age class (Adult vs. Juvenile)	6.361	3.901	1.631	0.1030	
		Age class (Adult vs. Subadult)	−0.239	1.858	−0.129	0.8975	
		Age class (Juvenile vs. Subadult)	−7.545	4.203	−1.795	0.0726	+
		Residency (Resident vs. Visitor)	0.196	1.862	0.105	0.9161	
		Residency (Resident vs. Vagrant)	−1.056	2.405	−0.439	0.6608	
		Residency (Visitor vs. Vagrant)	−1.676	2.432	−0.689	0.4906	
		Location : Feeding order	0.611	0.816	0.749	0.4538	
		Location : Sex	0.683	0.882	0.774	0.4392	
		Feeding order : Sex	−17.467	3785.431	−0.005	0.9963	
		Location : Feeding order : Sex	16.710	3785.431	0.004	0.9965	
	Feeding order (Final model)	(Intercept)	−9.076	2.146	−4.230	<0.0001	***
		Feeding order (First vs. Second)	−1.121	0.310	−3.616	0.0003	***
	Location + Feeding order	(Intercept)	−8.919	2.149	−4.151	<0.0001	***
		Location (Bears vs. Wild boars)	−0.362	0.333	−1.085	0.2779	
		Feeding order (First vs. Second)	−0.977	0.336	−2.910	0.0036	**
	Location + Feeding order + Sex	(Intercept)	−7.360	2.160	−3.408	0.0007	***
		Location (Bears vs. Wild boars)	−0.361	0.333	−1.084	0.2782	
		Feeding order (First vs. Second)	−0.976	0.336	−2.907	0.0036	**
		Sex (Female vs. Male) (Intercept)	−2.104	4.150	−0.507	0.6121	
	Location * Feeding order	(Intercept)	−8.854	2.157	−4.105	<0.0001	***
		Location (Bears vs. Wild boars)	−0.573	0.402	−1.426	0.1540	
		Feeding order (First vs. Second)	−1.463	0.613	−2.388	0.0170	*
		Location * Feeding order	0.700	0.728	0.962	0.3360	
	Feeding order + Sex	(Intercept)	−7.521	2.157	−3.486	0.0005	***
		Feeding order (First vs. Second)	−1.121	0.310	−3.614	0.0003	***
		Sex (Female vs. Male)	−2.100	4.155	−0.506	0.6132	
b) Call rate	Calling frequency * Landing frequency (Full model)	(Intercept)	3.171	3.445	0.920	0.357	
		Calling frequency	0.019	0.061	0.315	0.753	
		Landing frequency	−0.016	0.046	−0.342	0.732	
		Calling frequency : landing frequency	−0.0002	0.0008	−0.256	0.798	
	Landing frequency (Final model)	(Intercept)	4.963	0.470	10.553	<0.0001	***
		Landing frequency (Intercept)	−0.036	0.006	−5.833	<0.0001	***
		(Intercept)	4.021	0.932	4.313	<0.0001	***
	Calling frequency + Landing frequency	Calling frequency	0.004	0.003	1.169	0.2426	
		Landing frequency	−0.027	0.010	−2.677	0.0074	**
c) Call rate	Residency	(Intercept)	2.381	0.069	34.50	<0.0001	***
		Residency (Resident vs. Visitor)	−0.296	0.094	−3.152	0.0016	**
		Residency (Resident vs. Vagrant)	−0.621	0.182	−3.416	0.0006	***
		Residency (Visitor vs. Vagrant)	−0.325	0.181	−1.794	0.0728	+
	Focal Individual	Intercept	2.443	0.071	34.40	<0.0001	***
		Bi vs. Ge	−0.694	0.180	−3.853	0.0001	***
		Bi vs. Go	−0.438	0.206	−2.131	0.0331	*
		Bi vs. Hm	−0.495	0.165	−3.007	0.0026	**
		Bi vs. La	−0.550	0.133	−4.147	>0.0001	***
		Bi vs. Ti	−0.080	0.121	−0.658	0.5103	
		Ge vs. Go	0.255	0.256	0.998	0.3181	
		Ge vs. Hm	0.199	0.223	0.891	0.3731	
		Ge vs. La	0.144	0.199	0.720	0.4713	
		Ge vs. Ti	0.614	0.195	3.143	0.0017	**
		Go vs. Hm	−0.057	0.248	−0.288	0.8193	
		Go vs. La	−0.112	0.226	−0.495	0.6203	
		Go vs. Ti	0.359	0.218	1.646	0.0997	+
		Hm vs. La	−0.055	0.192	−0.289	0.0243	*
		Hm vs. Ti	0.495	0.165	3.007	0.0026	**
		La vs. Ti	0.415	0.184	2.252	0.0243	*

*Note*. (:) indicate interactions between fixed effects; (*) and (+) indicate significance levels

*** *p* < 0.001.

** *p* < 0.01.

* *p* < 0.05.

+ *p* < 0.1
